# Pediatric Traumas and Paradigm Shifts: The Necessary Adaption of the Steerable Microcatheter in Pediatric Interventional Radiology

**DOI:** 10.7759/cureus.4125

**Published:** 2019-02-23

**Authors:** Taylor S Harmon, Joanna Kee-Sampson, Timothy S Hester, Saeed Bashir, Jerry Matteo

**Affiliations:** 1 Radiology, University of Florida College of Medicine, Jacksonville, USA; 2 Radiology, University of Florida Health Shands Hospital, Gainesville, USA; 3 Surgery, University of Florida College of Medicine, Jacksonville, USA; 4 Interventional Radiology, University of Florida College of Medicine, Jacksonville, USA

**Keywords:** pediatric interventional radiology, steerable microcatheter, pediatric trauma, solid organ trauma, splenic artery embolization, renal artery embolization, blunt abdominal trauma, pelvic angiography, pseudoaneurysm, pushable coils

## Abstract

The use of traditionally available intra-arterial devices have historically been designed with the adult patient population in mind. Currently, there are not manufactured devices specifically tailored for use during pediatric interventional procedures, pressuring interventional operators to adapt commonplace and readily available devices for interventional management. Experienced interventional operators understand that pediatric and adult interventions can entail vastly different management, affecting patient care and outcomes. To address the pitfalls in pediatric interventional management, an accredited fellowship specifically for pediatric interventional radiology is available. However, devices must equally evolve with the training available in order to adequately address interventional management of the pediatric patient population. Interventional device innovation can be considered the initial step towards bridging the technical and procedural gaps necessary for refining pediatric intervention. The introduction of steerable microcatheters in interventional radiology has innovated procedural protocols, but has never been documented in pediatric patients until this time.

## Introduction

Pediatric trauma can often involve the vasculature or solid organs, requiring various imaging modalities for work-up and diagnosis. Much like the adult patient population, management for pediatric trauma can be algorithmic, based on the patient presenting history [1]. For patients that present with hemodynamic instability, internal hemorrhage may be of concern. Diagnostic contrast-enhanced angiography can be used to identify bleeding, and further interventional management can be performed if necessary. Likewise, solid organ injury can be the result of blunt force trauma in the pediatric population, in such case, a contrast-enhanced computed tomography (CT) of the abdomen and pelvis could direct interventional embolization if necessary. Though the adult and pediatric interventional protocols for procedures might be the same, the inter-arterial devices used for trauma in the pediatric population must be tailored for the lumen of smaller vasculature.

Solid organ arterial embolization has become a hallmark adjunctive interventional treatment in addition to the non-operative management of blunt trauma. In 1995, Sclafani et al. first described splenic embolization for blunt force trauma [2]. In that documented study, 150 hemodynamically stable patients with past medical histories of abdominal blunt force trauma, receiving nonoperative management (NOM), were screened for the possibility of receiving transcatheter splenic embolization. Sixty of the patients in the study met the inclusion criteria to receive splenic embolization intervention, which ultimately lead to a 98.5% splenic salvage rate; the efficiency of successful splenic embolization has yet to be matched [2-3].

Currently, splenic artery embolization remains the standard of care for blunt splenic trauma in patients that are hemodynamically stable. Since splenic embolization was first performed, a larger spectrum of high-grade blunt splenic trauma has been proven to be applicable for transcatheter embolization. In a large retrospective study, Bhullar et al. showed a strong correlation between contrast blush on angiography with active bleeding, and the necessity to perform splenic embolization in patients receiving NOM [4]. Furthermore, in the same study, it was demonstrated that patients with grade four or five blunt splenic trauma often failed NOM in the absence of contrast blush on angiography, because transcatheter embolization was not performed [4]. Therefore, the resulting data showed that high-grade splenic trauma should undergo splenic embolization regardless of contrast blush on angiography to maximize the success of NOM [4].

As previous data has shown that the utilization of transcatheter embolization for solid organ trauma is necessary in many scenarios, interventional operators have also refined techniques when performing these procedures. Currently, there are two types of splenic embolization which can be utilized in the trauma setting [5]. The first is proximal splenic artery embolization, which is indicated in hemodynamically stable patients with one or more of the following contrast-enhanced CT findings: American Association for the Surgery of Trauma splenic injury grade of three or higher, contrast extravasation, moderate to large hemoperitoneum, arteriovenous fistula, or pseudoaneurysm [5]. The second is distal splenic branch embolization, which includes the embolization of selective splenic branches beyond the proximal splenic artery. Indications for such may include positive angiographic findings such as arteriovenous fistula, pseudoaneurysm formation, and contrast extravasation [5]. Distal splenic branch embolization entails the use of a microcatheter system which promotes an increase in procedural time, contrast material, and radiation exposure [5].

The three anticipated risks that include increased procedural time, contrast material exposure, and radiation exposure in relation to distal splenic branch embolization, can all be addressed with the efficient use of steerable microcatheters by interventional operators. Additionally, the inherent ability of steerable microcatheters to access smaller vascular lumen, make these devices an optimal choice when performing pediatric interventions. To reduce these risks that may arise as a result of transcatheter embolization, the added maneuverability of steerable microcatheters can be used when valuing procedural efficiency. The following cases demonstrate the adaptability of the steerable microcatheter, applied to a proximal splenic artery embolization, pelvic angiography, and renal artery branch embolization in two pediatric patients.

## Technical report

The following two cases will demonstrate the efficiency of a steerable microcatheter system in the setting of two separate pediatric traumas. A SwiftNINJA® steerable microcatheter (Merit Medical Systems, South Jordan, Utah) was used in both procedural settings to selectively catheterize arterial branch, otherwise more arduous for a traditional wire and catheter system.

Pediatric splenic artery embolization

**Figure 1:**
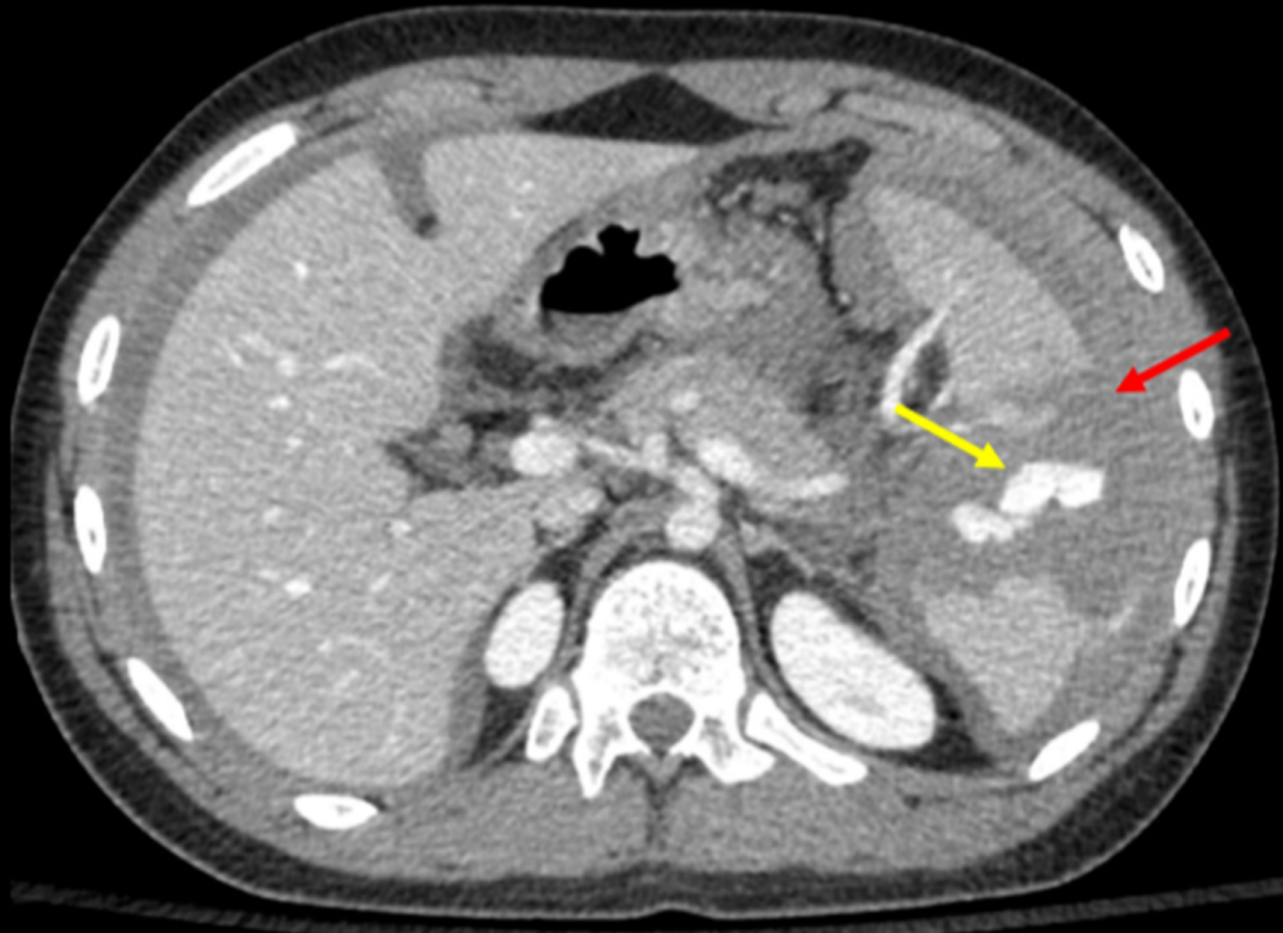
Initial Contrast-enhanced Computed Tomography of Splenic Injury The contrast-enhanced computed tomography of the patient's spleen shows a splenic injury, as a result of blunt force trauma. The yellow arrow shows active contrast extravasation, and the red arrow shows a hematoma.

A hemodynamically stable 14-year-old male without a significant past medical history presented with blunt abdominal trauma. A contrast-enhanced CT showed a grade three blunt splenic injury with hemorrhage (Figure 1).

In addition to medical management, interventional radiology was consulted for the possibility of a splenic artery embolization.

**Figure 2:**
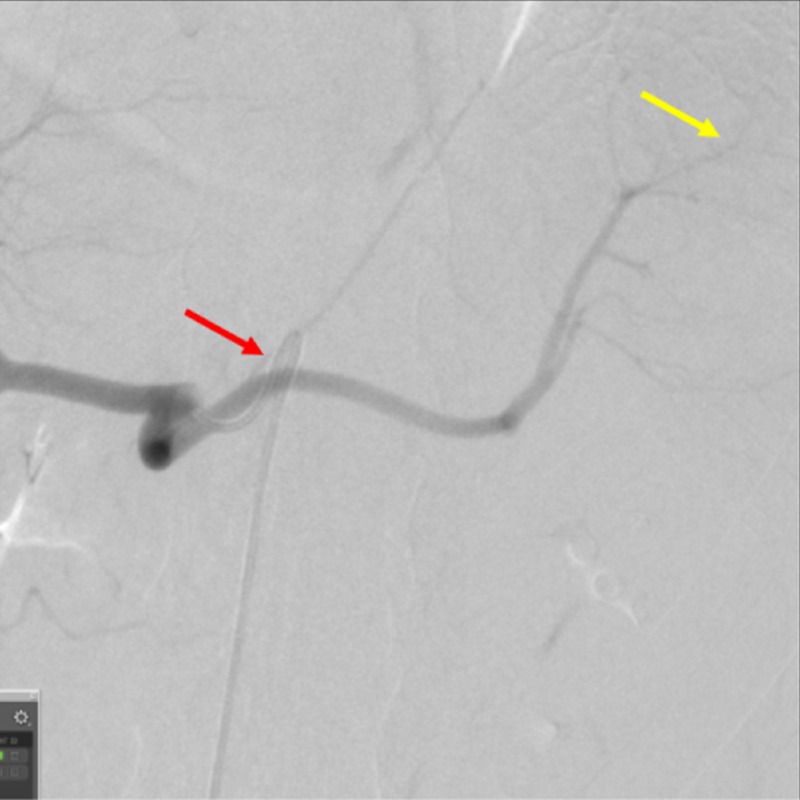
Catheterization of the Celiac Artery The angiogram demonstrates a widely patent celiac artery. The red arrow shows the Mickelson catheter, and the yellow arrow shows the segmental branches of the splenic artery.

After obtaining informed consent, the patient was taken to an interventional radiology suite, and prepared on an angiography table. Femoral arterial access was obtained within the patient’s right groin using a micropuncture system, and subsequently upgraded to a five French sheath. Under fluoroscopic guidance, a Benston catheter was introduced into the abdominal aorta. Over the guidewire, a Mickelson catheter was introduced, and the celiac artery was selectively catheterized. Hand injected contrast images were obtained, and showed the celiac artery to be widely patent (Figure 2).

**Figure 3:**
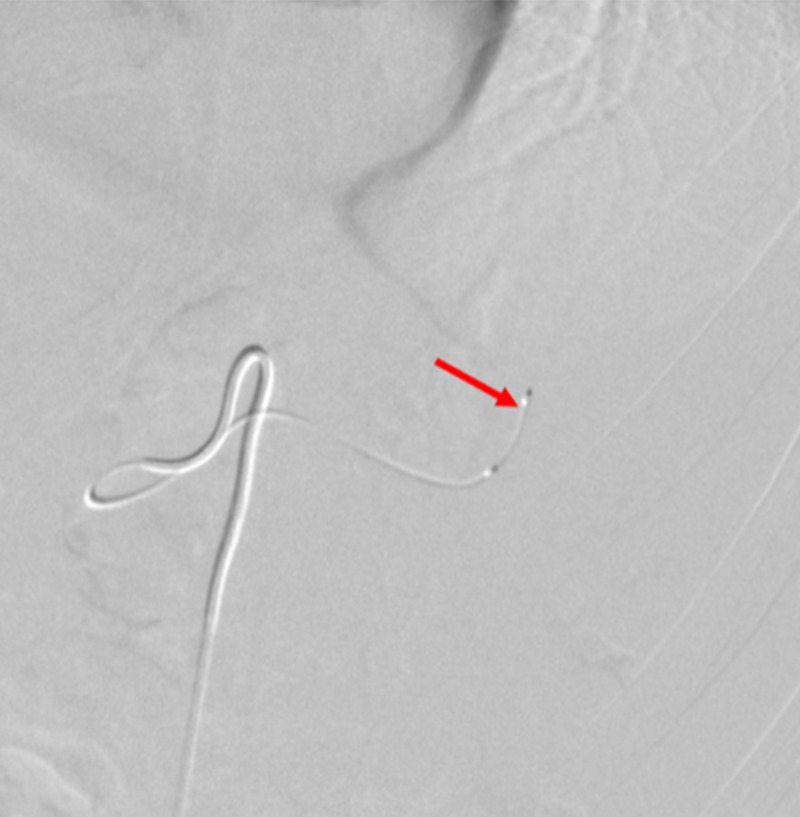
Advancement of a SwiftNINJA® Steerable Microcatheter Through the Mickelson Catheter A SwiftNINJA® steerable microcatheter (red arrow) is seen as it is advanced into the splenic artery through the Mickelson catheter.

A SwiftNINJA® steerable microcatheter was selectively advanced into the splenic artery quickly without the use of a guidewire (Figure 3).

**Figure 4:**
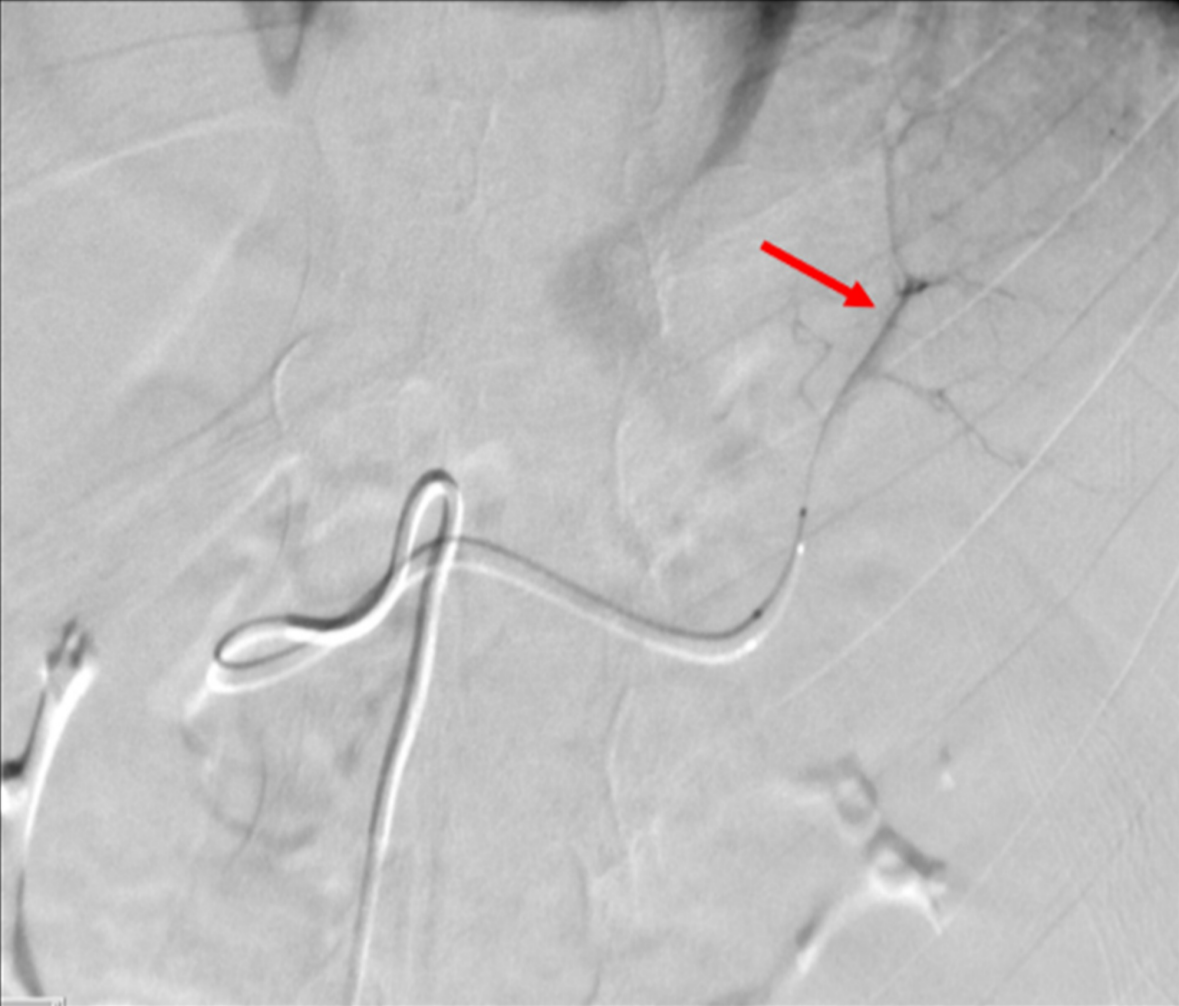
Angiography of the Mid to Distal Splenic Artery Demonstrating Pruning of Small Caliber Vessels The follow-up angiogram demonstrates vasospasm of the mid to distal splenic artery. The red arrow shows small vessel pruning, an intrinsic mechanism of response to blunt organ injury.

Additional contrast images were obtained, demonstrating pruning of small caliber vessels as would be seen of blunt organ injury in adolescence (Figure 4).

**Figure 5:**
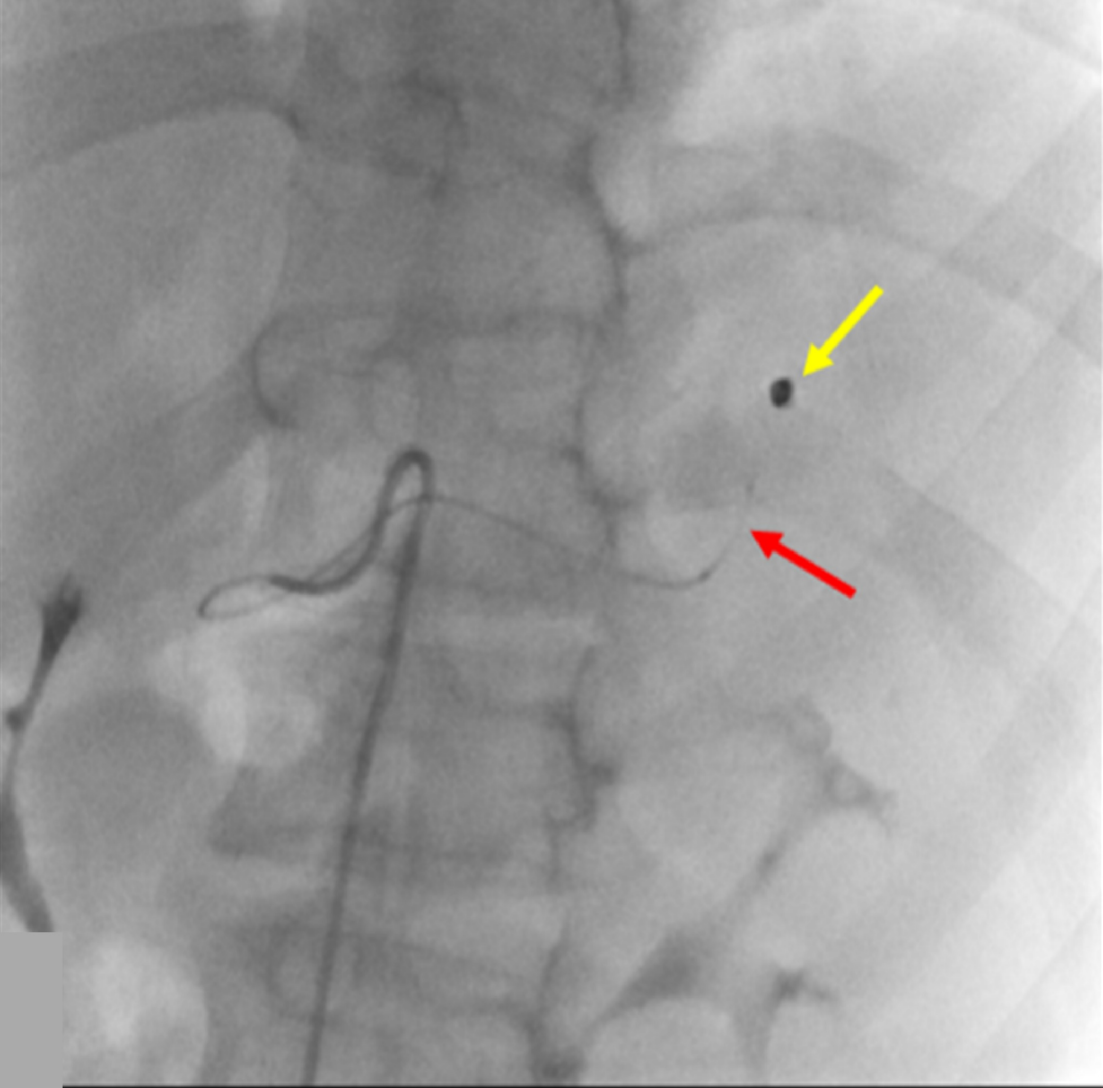
Embolization of the Splenic Artery The angiogram shows the SwiftNINJA® steerable microcatheter (red arrow) depositing the first pushable coil (yellow arrow) during the splenic artery embolization.

The decision was made by the interventional operator to perform a splenic artery embolization, due to the high-grade splenic injury. Embolization of the splenic artery was performed using five pushable coils through the SwiftNINJA® steerable microcatheter (Figure 5).

**Figure 6:**
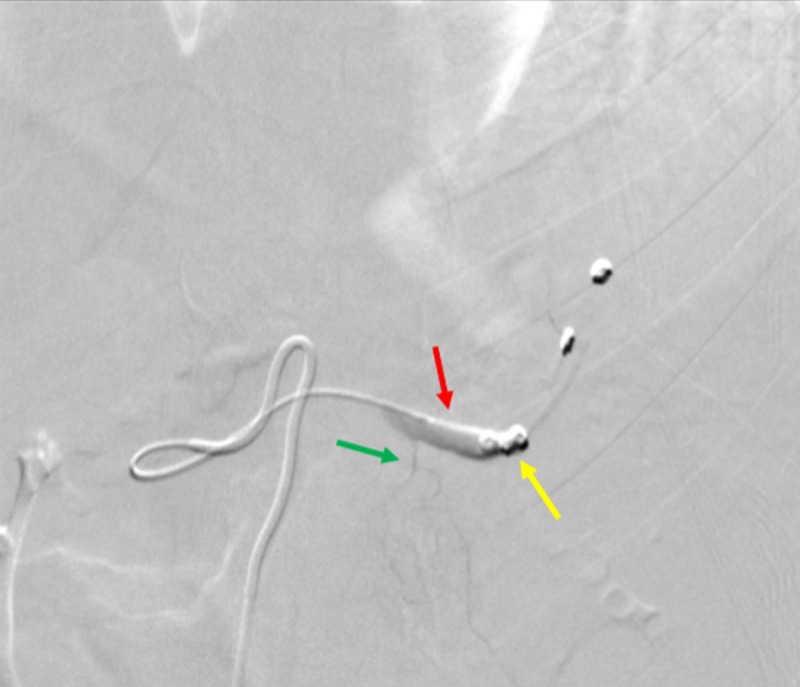
Post-procedural Angiography of the Splenic Artery Embolization A final angiogram shows a successful splenic artery embolization. Cessation of flow is seen through the splenic artery at the tip of the SwiftNINJA® steerable microcatheter (red arrow), where the last pushable coils were deposited (yellow arrow). The green arrow shows that there is still blood flow through the dorsal pancreatic artery, allowing the pancreas to continue being perfused.

Follow-up angiography showed cessation of flow within the main splenic artery, and persistent blood flow within the dorsal pancreatic artery (Figure 6).

A right femoral arteriogram was performed through the sheath indicating the patient was an adequate candidate for a closure device. All wires, catheters, and sheaths were removed, and the patient’s right groin site was closed with an Angio-Seal® (Terumo Medical Corporation, Somerset, New Jersey) closure device. The patient tolerated the procedure well without any immediate complications.

Pediatric renal artery embolization

**Figure 7:**
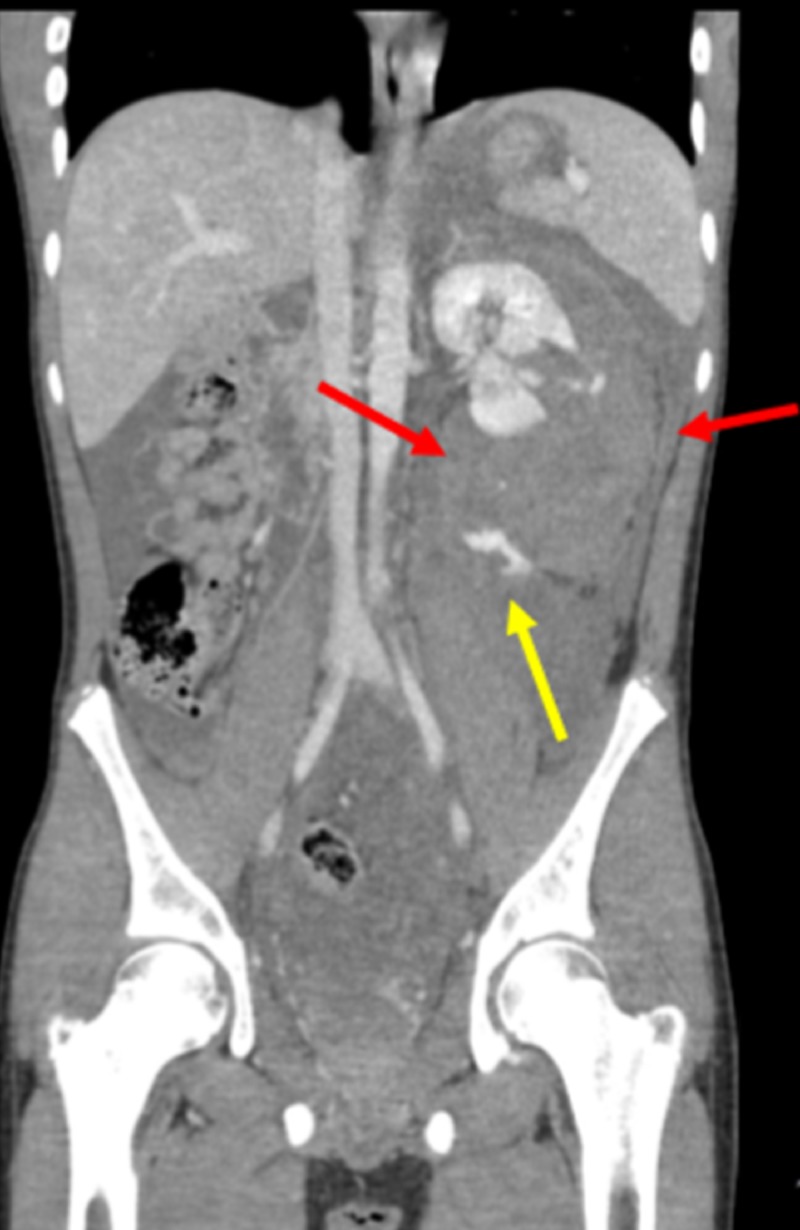
Initial Contrast-enhanced Coronal Computed Tomography of the Abdomen and Pelvis The initial contrast-enhanced coronal computed tomography of the patient shows a perinephric hematoma near the left kidney (borders of the hematoma are shown by the red arrows). The yellow arrow shows active contrast extravasation indicating hemorrhage.

A 14-year-old male with a past medical history of a traumatic perinephric hematoma of the left kidney, presented for possible pelvic and renal artery embolization (Figure 7).

**Figure 8:**
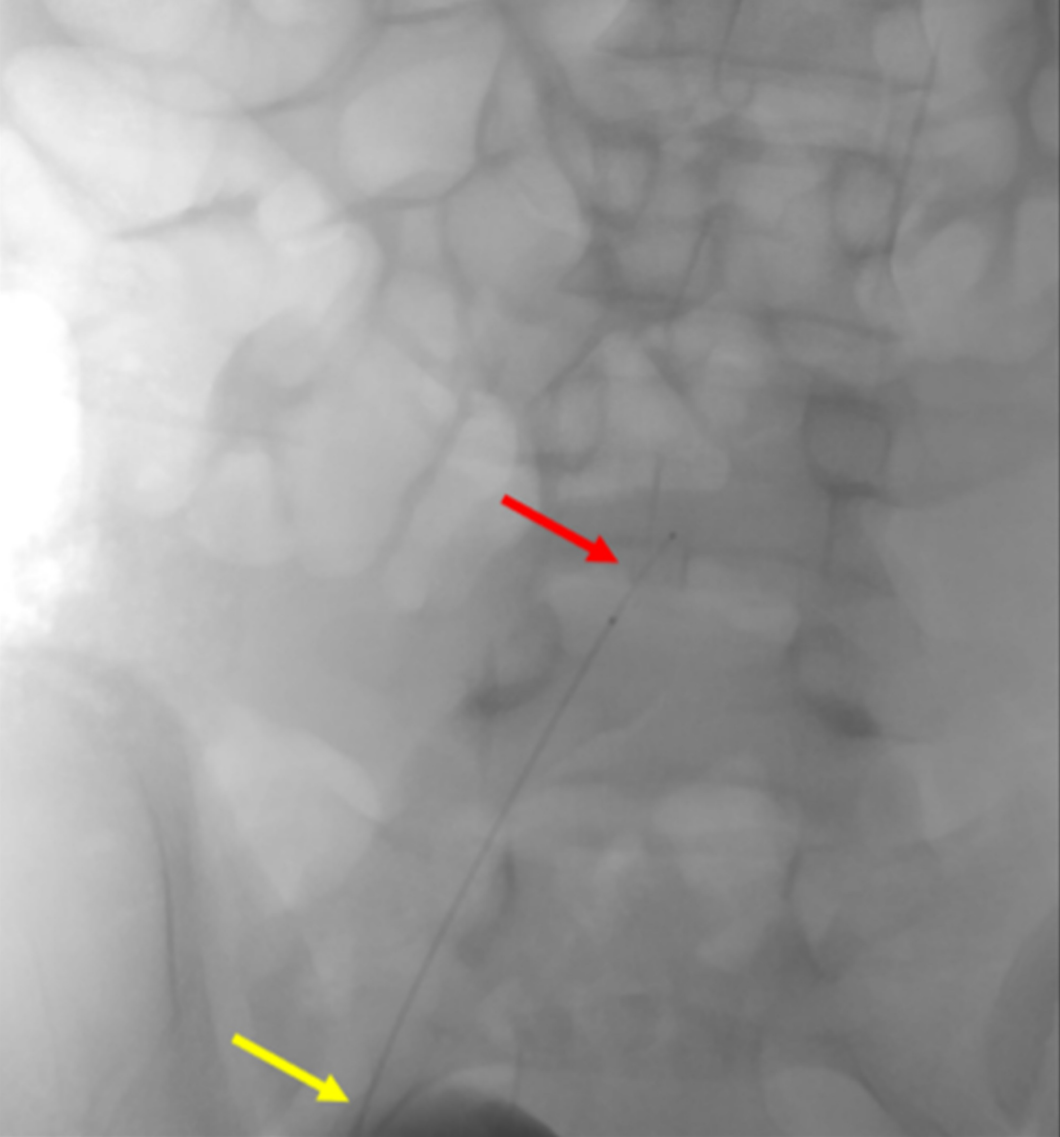
Advancement of the SwiftNINJA® Steerable Microcatheter Through the Sheath The advancement of the SwiftNINJA® steerable microcatheter (red arrow) can be seen through the sheath (yellow arrow).

After obtaining informed consent, the patient was taken to an interventional radiology suite, and prepared on an angiography table. Femoral arterial access was obtained within the patient’s right groin using a micropuncture system, and subsequently upgraded to a four French sheath. A SwiftNINJA® steerable microcatheter was advanced within the sheath without the use of a guidewire (Figure 8).

**Figure 9:**
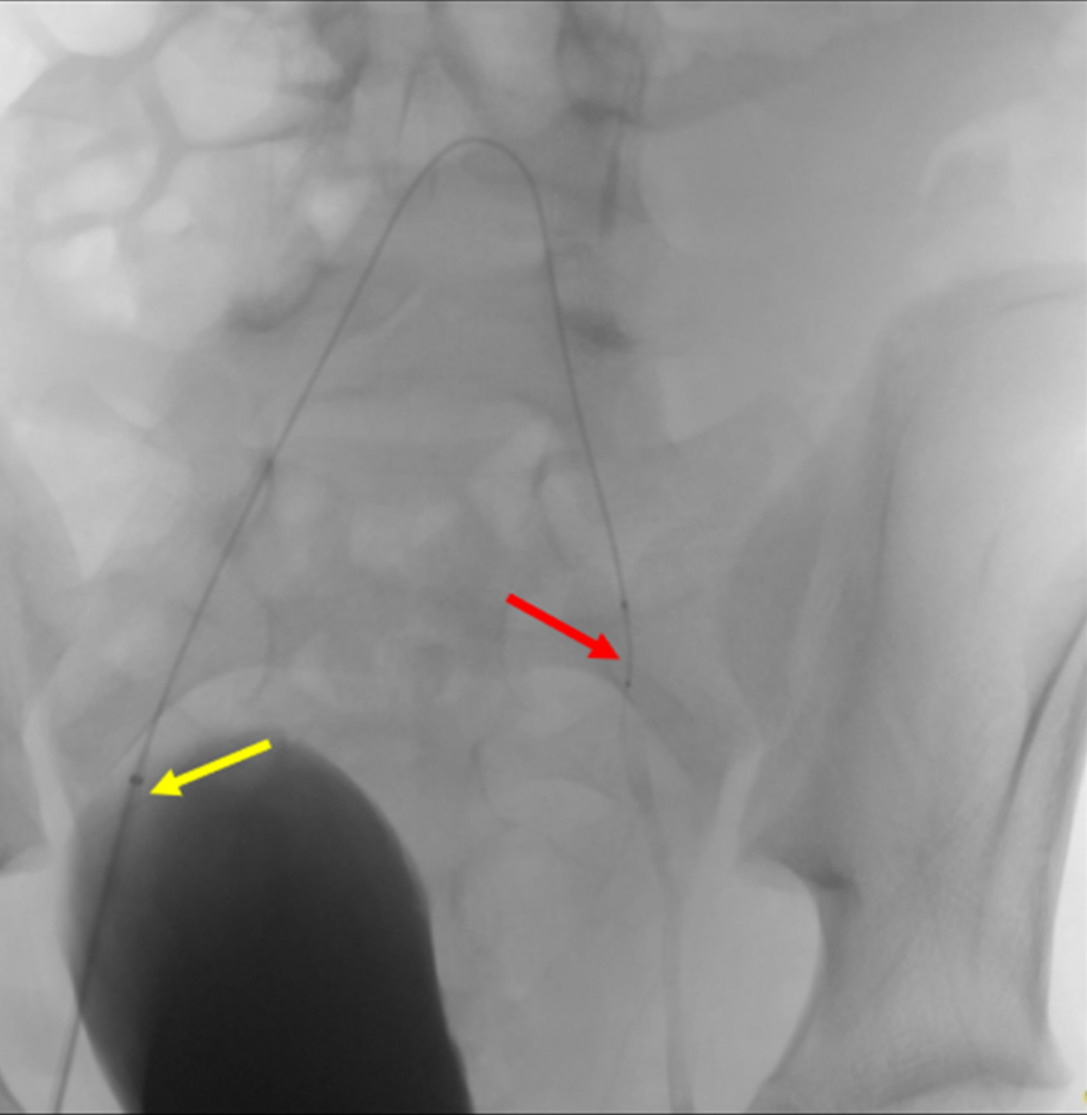
Quick Access of the Left Internal Iliac Artery Using a SwiftNINJA® Steerable Microcatheter The figure shows the SwiftNINJA® steerable microcatheter accessing the patient's left internal iliac artery (red arrow), without the use of a guidewire. The yellow arrow shows the sheath that the SwiftNINJA® steerable microcatheter is advanced from.

The SwiftNINJA® steerable microcatheter was quickly navigated to the left internal iliac artery (Figure 9).

**Figure 10:**
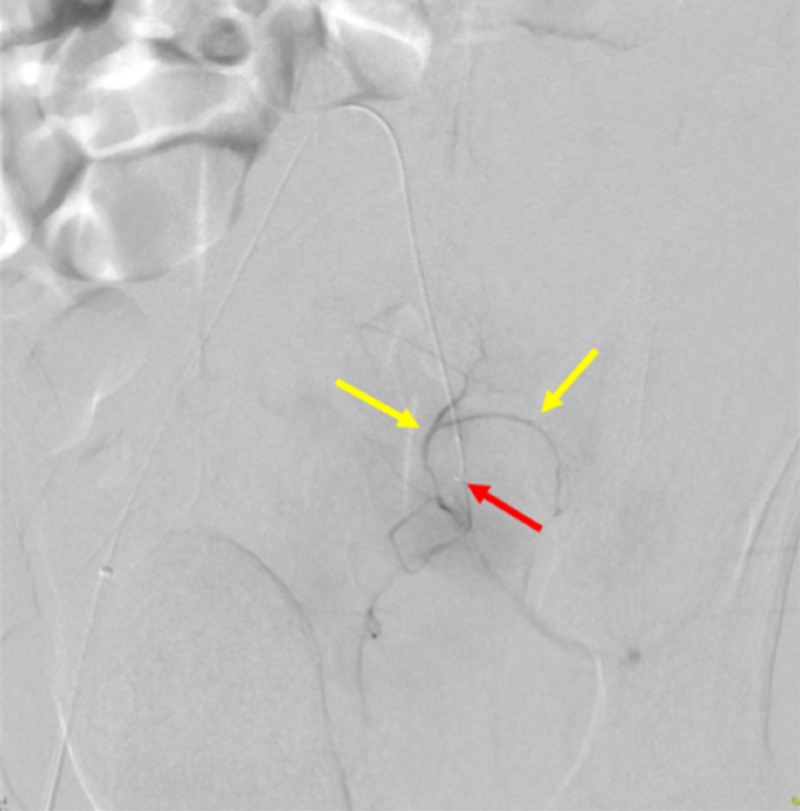
Angiogram Through the SwiftNINJA® Steerable Microcatheter Demonstrating the Absence of Contrast Extravasation An angiogram of the patient's left internal iliac artery was done through the SwiftNINJA® steerable microcatheter (red arrow), demonstrating the absence of contrast extravasation (yellow arrow), and negating the need for pelvic embolization.

A subsequent angiogram through the SwiftNINJA® demonstrated no evidence of contrast extravasation (Figure 10).

**Figure 11:**
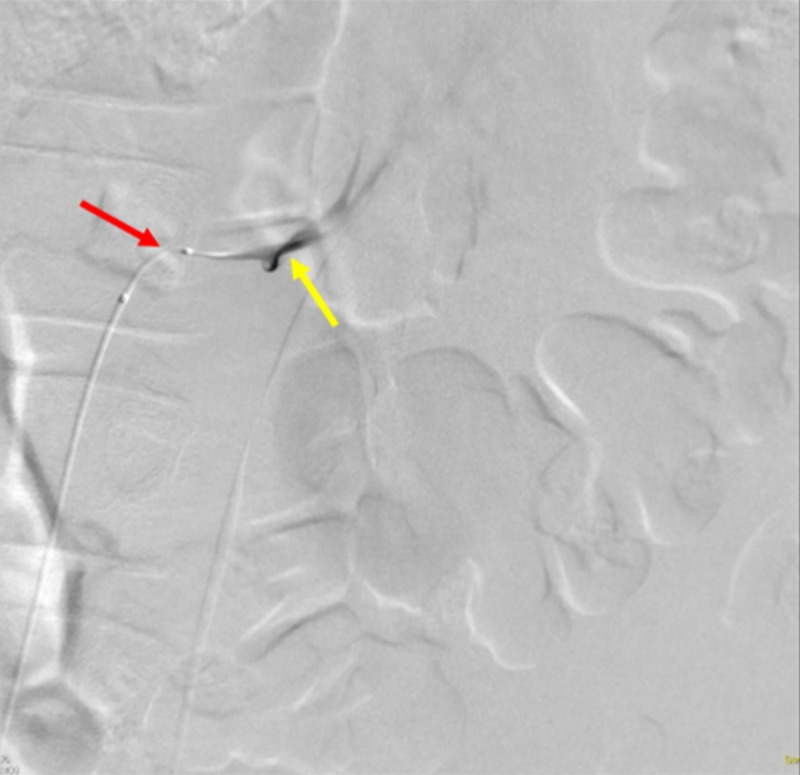
Hand Injected Contrast Angiogram Showing Vessel Cut Off in the Left Renal Artery A hand injected contrast angiogram through a SwiftNINJA® steerable microcatheter (red arrow) shows vessel cut off (yellow arrow) in the left renal artery, indicating transection.

The left renal artery was selectively catheterized with a SwiftNINJA® steerable microcatheter, and hand injected contrast images were obtained, which demonstrated the left renal artery to be widely patent. At that time, it was revealed that evidence of contrast extravasation was due to a vessel cut off (Figure 11).

**Figure 12:**
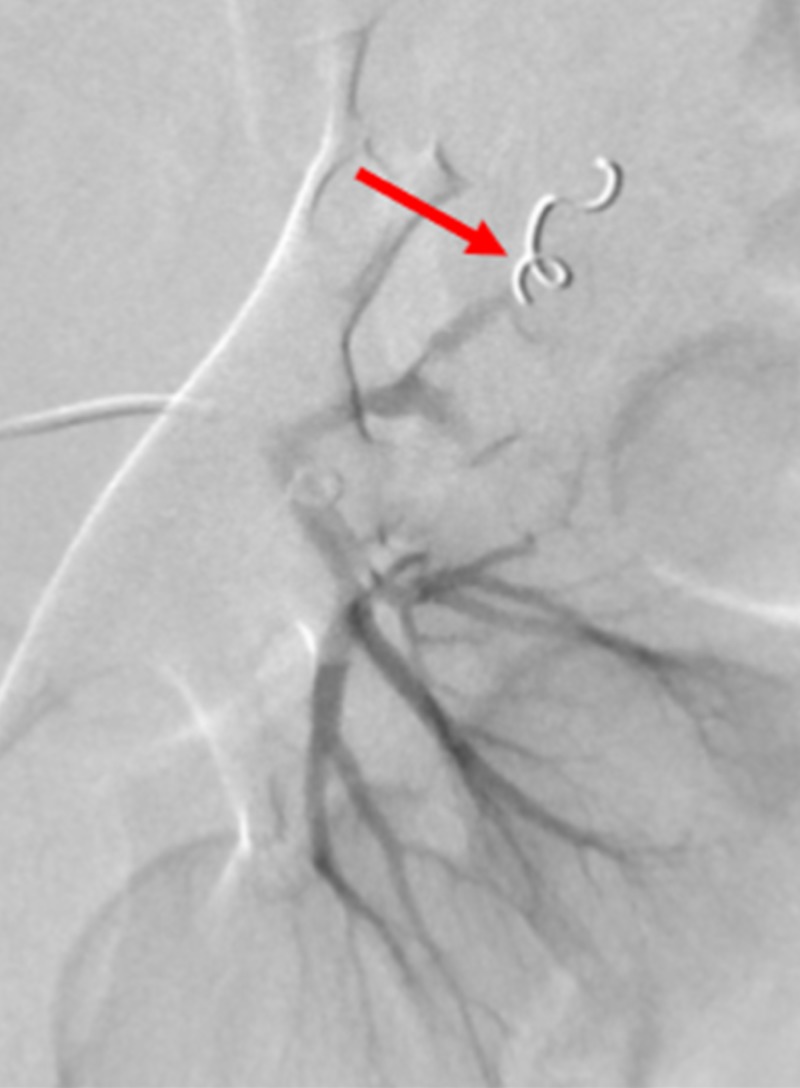
Cook® Hilal Pushable Coils Used to Embolize Segmental Branches of the Left Renal Artery A 3 mm by 3 cm Cook® Hilal pushable coil (red arrow) is seen after deployment through the SwiftNINJA® steerable microcatheter, in a successful attempt to embolize the patient's upper mid-pole segmental branch of the left renal artery.

A SwiftNINJA® steerable microcatheter was used to quickly and selectively catheterize the mid-upper pole branch left renal artery, without the use of a guidewire. A 3 mm by 3 cm Cook® Hilal pushable coil (Cook Medical, Bloomington, Indiana) was deposited through the SwiftNINJA® steerable microcatheter, leading to embolization. A different upper mid-pole segmental branch of the left renal artery was selected with the SwiftNINJA® steerable microcatheter, and two more 3 mm by 3 cm Cook® Hilal pushable coils were deposited within this upper mid-pole segmental branch, due to evidence of contrast extravasation (Figure 12).

Angiographic contrast images were subsequently obtained, demonstrating no further filling defects. Additional hand contrast injections within the left main renal artery demonstrated preservation of flow to the superior and inferior poles of the patient’s left kidney.

The sheath, wires, and catheters were removed, and manual pressure was held at the patient’s groin site. A sterile dressing was placed after hemostasis was ensured. The patient tolerated the procedure well without any immediate complications.

## Discussion

Though not necessarily developed for the pediatric patient population, steerable microcatheters have been documented as efficient devices that allow interventional operators to perform a broad spectrum of procedures in less time and with less radiation exposure to the patient [5]. Intrinsically, the steerable microcatheter is operator guided and can be directed without the use of a traditional wire. As a result, steerable microcatheters supply added maneuverability, providing an advantage over the traditional wire and catheter system [5].

The article 'Welcome to the New Era: A Completely Wireless Interventional Procedure' described the first vascular interventional application of the steerable microcatheter, which also disclosed the reduced procedural time [5]. Others have reported the surpassing functionality of steerable microcatheters over traditional wire and catheter systems in the extravascular setting [6-7]. Harmon et al. first recognized how steerable microcatheters show promise in the reduction of radiation exposure and procedural time in patients with acute ischemic stroke [8]. For the first time, the preceding cases show how the innovation of steerable microcatheters in the adult population can be applied in pediatric interventional radiology.

Pediatric interventional radiology originated immediately behind the interventional management of the adult population. As a result of the development of interventional practices and techniques, pediatric interventional radiology is much more common. Moreover, because of the obvious need in the general patient population, especially in the setting of pediatric trauma, pediatric interventional radiology has been evolving to meet these demands. The preceding cases demonstrate the first documented evidence for the efficiency of a steerable microcatheter system in pediatric patients, but could also allude to the departure from the traditional wire and catheter system altogether, ushering in a new era of interventional radiology.

## Conclusions

Pediatric interventional radiology is a prominent and emerging subspecialty that deserves the same attention granted to the adult interventional patient population. The preceding cases display the efficiency that the steerable microcatheter has over a traditional wire and catheter system, in the setting of pediatric trauma. Likewise, steerable microcatheter systems show a clear advantage in the pediatric patient population, which is both authentic and predictive of the prospective future for interventional radiology.
